# Supplementary material for: Expression of CD47 and SIRPα Macrophage Immune-Checkpoint Pathway in Non-Small-Cell Lung Cancer
Source: Cancers (Basel). 2022 Apr 1;14(7):1801. doi: 10.3390/cancers14071801 (PMC8997641; doi:10.3390/cancers14071801)
Supplement: Supplementary file 1 [file cancers-14-01801-s001.zip › cancers-1661745-supplementary.pdf]

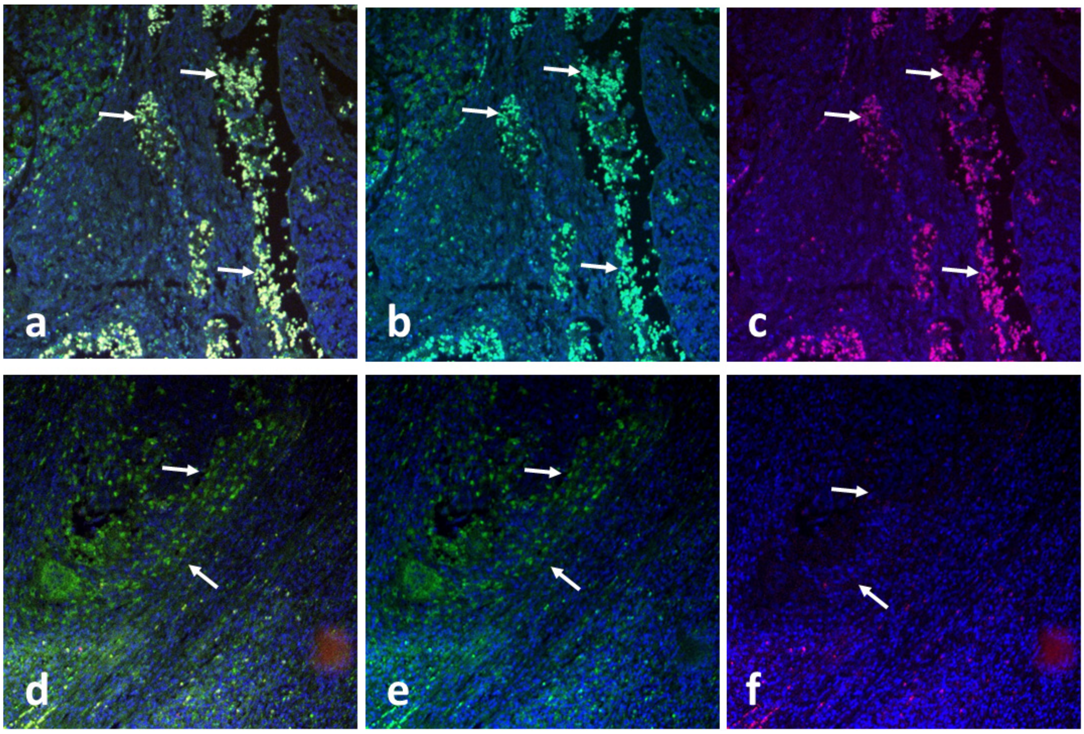

Supplemental Figure S1. Triple CD68/SIRPα immunofluorescence with Hoechst 33,342 (blue staining of cell nuclei) confocal images of lung cancer tissue. White arrows show the areas of macrophage infintration. (**a**–**c**) A cancer tissue section showing intense tumor stroma infiltration by CD68+ (green; **b**) and SIRPα+ (red; **c**) macrophages, with extensive co-localization (yellow; **a**); white arrows. (**d**–**f**) A cancer tissue section showing intense tumor stroma infiltration by CD68+ (green; **e**), scarce presence of SIRPα+ macrophages (red; **f**), and lack scarce co-localization (yellow; **d**); white arrows.
